# Optimization of Ultrasonic-Assisted Extraction of α-Glucosidase Inhibitors from *Dryopteris crassirhizoma* Using Artificial Neural Network and Response Surface Methodology

**DOI:** 10.3390/metabo13040557

**Published:** 2023-04-13

**Authors:** Nguyen Viet Phong, Dan Gao, Jeong Ah Kim, Seo Young Yang

**Affiliations:** 1 Vessel-Organ Interaction Research Center, VOICE (MRC), College of Pharmacy, Kyungpook National University, Daegu 41566, Republic of Korea; ngvietphong@gmail.com; 2 BK21 FOUR Community-Based Intelligent Novel Drug Discovery Education Unit, College of Pharmacy and Research Institute of Pharmaceutical Sciences, Kyungpook National University, Daegu 41566, Republic of Korea; 3 Institute of Chinese Materia Medica, China Academy of Chinese Medical Sciences, Beijing 100700, China; dgao@icmm.ac.cn; 4 Department of Pharmaceutical Engineering, Sangji University, 83 Sangjidae-gil, Wonju 26339, Republic of Korea

**Keywords:** *Dryopteris crassirhizoma*, phloroglucinol, α-glucosidase, response surface methodology, artificial neural network

## Abstract

*Dryopteris crassirhizoma* Nakai is a plant with significant medicinal properties, such as anticancer, antioxidant, and anti-inflammatory activities, making it an attractive research target. Our study describes the isolation of major metabolites from *D. crassirhizoma*, and their inhibitory activities on α-glucosidase were evaluated for the first time. The results revealed that nortrisflavaspidic acid ABB (**2**) is the most potent α-glucosidase inhibitor, with an IC_50_ of 34.0 ± 0.14 μM. In addition, artificial neural network (ANN) and response surface methodology (RSM) were used in this study to optimize the extraction conditions and evaluate the independent and interactive effects of ultrasonic-assisted extraction parameters. The optimal extraction conditions are extraction time of 103.03 min, sonication power of 342.69 W, and solvent-to-material ratio of 94.00 mL/g. The agreement between the predicted models of ANN and RSM and the experimental values was notably high, with a percentage of 97.51% and 97.15%, respectively, indicating that both models have the potential to be utilized for optimizing the industrial extraction process of active metabolites from *D. crassirhizoma*. Our results could provide relevant information for producing high-quality extracts from *D. crassirhizoma* for functional foods, nutraceuticals, and pharmaceutical industries.

## 1. Introduction

*Dryopteris crassirhizoma* Nakai is a semi-evergreen plant that is widely distributed in China, South Korea, and Japan; the root of this plant is used in the Chinese Pharmacopoeia to cure viral disease and verminosis [1]. Recent studies revealed many pharmacological effects of this plant such as anti-cancer, antioxidant, anti-inflammatory, protein tyrosine phosphatase 1B (PTP1B), and *β*-glucuronidase inhibitory activities [2,3,4,5,6]. Previous phytochemical investigations of the rhizomes of *D. crassirhizoma* demonstrated the presence of various components, including phloroglucinols, flavonoids, and triterpenes [5,6]. Phloroglucinols, principal chemical constituents of the isolate from *D. crassirhizoma*, exhibit a multitude of pharmacological activities. Monomeric phloroglucinol butyryl-3-methylphloroglucinol was found to exhibit potent inhibition of platelet aggregation induced by collagen and arachidonic acid, with ratios of 92.36% and 89.51%, respectively [7]. Dimeric compounds dryoptol G and 3″-*epi*-dryoptol G exhibited significant inhibitory effects on the production of nitric oxide and the secretion of inflammatory cytokines interleukin-1 beta (IL-1β) and interleukin-18 (IL-18) in RAW264.7 cells induced with lipopolysaccharide [8]. Trimeric phloroglucinol filixic acid ABA has been identified as responsible for the antiviral effect against influenza A virus H1N1 [8]. Additionally, both filixic acid ABA and dryocrassin ABBA were shown to have dose-dependent inhibitory activity against SARS-CoV-2 infection in Vero cells using immunofluorescence-based antiviral assays [9]. Especially, flavaspidic acid AP and nortrisflavaspidic acid ABB are two phloroglucinols that have been previously identified in *D. crassirhizoma* and characterized. The investigations of their biological activity have revealed significant xanthine oxidase inhibitory, skin-whitening, and anti-influenza virus (H5N1) effects [10,11,12]. Moreover, these compounds are considered unique quality control indices for *D. crassirhizoma*, which may be used as reliable markers for evaluating the plant’s quality and consistency [12]. The diverse applications in functional foods, nutraceuticals, and the pharmaceutical industry highlight their commercial value and thus make them attractive targets for additional research and development. Unfortunately, there is no standardized set of optimum conditions for extracting these compounds from *D. crassirhizoma*. Therefore, to make the best use of this plant resource, the extraction yield of these substances should be maximized.

Recently, ultrasonic-assisted extraction (UAE) has been demonstrated to be an environmentally friendly, productive, inexpensive, simple, and green extraction method [13]. Furthermore, compared with traditional extraction methods, such as maceration, Soxhlet, and percolation, UAE shows a higher recovery of bioactive components and requires a lower amount of organic solvent, lower temperature, and shorter extraction time. However, UAE is a complex process affected by the following factors: extraction time, solvent-to-material ratio, extraction temperature, and sonication power [14]. These factors may individually or jointly affect the extraction efficiency of bioactive components [15]. Experimental evaluation of the effects of different factors on the efficiency of UAE and optimization of the extraction conditions is an expensive and tedious process. Moreover, novel multivariate analysis methodologies, such as artificial neural network (ANN) and response surface methodology (RSM), may be used to confirm the independent and interactive effects of these factors and optimize extraction conditions. RSM, a commonly used statistical tool in analytical optimization, can examine the effect of single or multiple variables on the target response by developing a quadratic equation [13,16]. ANN is a new popular non-linear computational modeling method that demonstrates satisfactory capability and flexibility in data fitting, optimization, and prediction; thus, it has been used as a complementary model with RSM for the optimization of the extraction of bioactive components in chemical engineering [17,18,19]. To date, a comparison between ANN and RSM concerning the optimization of the extraction bioactive components from *D. crassirhizoma* has not been reported.

Therefore, the individual and interactive effects of the principal UAE parameters (solvent-to-material ratio, sonication time, and power) on target responses were analyzed. ANN and RSM were compared regarding the optimization of the UAE of *D. crassirhizoma* for maximizing the yield of bioactive components. Furthermore, for the first time, the inhibitory activity of *D. crassirhizoma* against α-glucosidase was evaluated.

## 2. Materials and Methods

### 2.1. Experimental Procedures

Ultrasonic extraction was conducted using Mujigae SD-350H ultrasonic bath (40 kHz, 0–400 W; Sungdong Ultrasonic, Seoul, Korea). Nuclear magnetic resonance (NMR) spectra (^1^H and ^13^C-NMR) were measured on a Bruker AVANCE 500 MHz system (Bruker, Karlsruhe, Germany) using tetramethylsilane as the internal standard, at 294 K. Column chromatography (CC) was conducted on Millipore silica gel 60 (0.040–0.063 mm; Merck, Darmstadt, Germany) and Cosmosil 75C_18_-PREP gel (Nacalai Tesque, Kyoto, Japan). Preparative high-performance liquid chromatography (HPLC) was performed using an HPLC Water system (Waters, Middleton, USA) with a YMC-Pack ODS column (10 mm × 250 mm, 5 μm). α-Glucosidase (G5003), 4-nitrophenyl-D-glucopyranoside (N1377), and acarbose (A8980) were purchased from Merck (Germany).

### 2.2. Plant Material

The rhizomes of *D. crassirhizoma* were purchased from a local market at Jeonju-si, Korea, in September 2019 and authenticated by Professor Byung Sun Min, Daegu Catholic University. A voucher specimen (accession code 23A–DC) was maintained in the Laboratory of Pharmacognosy, College of Pharmacy, Kyungpook National University.

### 2.3. Extraction and Isolation

The dried rhizomes of *D. crassirhizoma* (1.0 kg) were extracted three times with ethanol (20 L × 4 h) under sonication. The extract was concentrated under reduced pressure, and the obtained ethanol residue (150 g) was suspended in water and partitioned with CH_2_Cl_2_ and ethyl acetate (EtOAc), yielding a CH_2_Cl_2_ extract (90.5 g) and EtOAc extract (89.9 g), respectively. The EtOAc extract was fractionated over silica gel CC using a stepwise eluent of CH_2_Cl_2_–acetone (gradient 20:1–1:1, *v*/*v*) and then CH_2_Cl_2_–MeOH (gradient 10:1–5:1, *v*/*v*) to obtain six fractions, A–F. Compound **1** (19.0 mg) was isolated from fraction B (5.1 g) using silica gel CC eluted with CH_2_Cl_2_–MeOH–H_2_O (6:1:0.1, *v*/*v*) and then purified by RP-18 CC eluting with MeOH–H_2_O (2:1, *v*/*v*). Compound **2** (10.1 mg) was isolated from fraction C (4.1 g) by reserved-phase C18 CC using MeOH–H_2_O (4:1, *v*/*v*) as the eluent, followed by preparative HPLC (isocratic mixture solvent 66% MeOH in distilled H_2_O, 6 mL/min over 60 min). Both ^1^H and ^13^C-NMR spectra of compounds **1** and **2** were obtained using the Bruker NMR spectrometer.

### 2.4. Evaluation of the Inhibitory Activity against α-Glucosidase

The α-glucosidase assay was performed as previously described [20]. First, 130 μL of α-glucosidase (0.16 unit/mL) in 0.1 mM phosphate buffer (pH 6.8) was added to a 96-well plate with 20 μL of test compounds dissolved in dimethylsulfoxide. Then, 50 μL of 1 mM substrate (4-nitrophenyl-D-glucopyranoside) in the buffer was added to the mixture. The intensity of *p*-nitrophenol converted from 4-nitrophenyl-D-glucopyranoside by α-glucosidase was measured after 15 min at 405 nm using Infinite 200 PRO spectrophotometer (Tecan, Zürich, Switzerland).

(1) $$\mathrm{Inhibitory}\;\mathrm{activity}\;\mathrm{rate}\;(\%)=\left[\frac{\left(\Delta C-\Delta S\right)}{\Delta C}\right]\times 100$$

where ΔC and ΔS are the intensity of control and inhibitor after 20 min, respectively. Acarbose was then selected as a positive control [21]. The inhibitory effect was elucidated via triplicate independent experiments. The results are shown as the mean ± standard error (SEM). Statistical differences were investigated using one-way ANOVA and Duncanʹs test with a *p*-value < 0.05. SigmaPlot 10.0 (Systat Software Inc., San Jose, CA, USA) was used to analyze the inhibition results.

### 2.5. HPLC Analysis

The *D. crassirhizoma* ethanol extract was analyzed using HPLC with a photodiode array detector (LC-20AD system, Shimadzu, Kyoto, Japan). Chromatographic separation was performed using an OptimaPak C_18_ column (RStech, Daejeon, Korea; 4.6 mm × 250 mm, 5 μm) at a flow rate of 1 mL/min. The mobile phase comprising 0.1% aqueous formic acid (solvent A) and acetonitrile (solvent B) at a linear-gradient elution program was: 0 min, 50% B; 30 min, 90% B; 60 min, 100% B. The wavelength for detection was 280 nm.

### 2.6. Selection of Variables

In this context, three extraction factors, including sonication time (min), sonication power (W), and solvent-to-material ratio (mL/g), were examined in the preliminary analysis. The total peak area of the bioactive components was selected as the response variable (Figure S1).

### 2.7. Box–Behnken Design for Optimization

Box–Behnken design (BBD) experiment was used to examine the interaction between the factors and determine the optimum operating conditions. Based on the results of single-factor experiments, the bounds of the variables were selected as 80–120 min for sonication time, 240–400 W for sonication power, and 40:1–120:1 for solvent-to-material ratio. A three-level [higher (+1), middle (0), and lower (−1)] and a three-factor approach were used for the experiment design, model construction, and data interpretation. A total of 17 independent experimental runs were conducted to evaluate the effect on the bioactive compounds and predict the optimum operating parameters. The results for three independent variables were optimized by RSM using Design-Expert 12 (Stat-Ease, Minneapolis, MN, USA).

### 2.8. ANN

The ANN was realized in MATLAB R2020b (MathWorks, Natick, MA, USA). A combined dataset comprising 17 data points was compiled, and the operating parameters of extraction were used as independent input variables. The feedforward backpropagation network was selected along random data division, Levenberg–Marquardt (trainlm) was selected as a training function, and mean squared error (MSE) was used as a performance function with one hidden and one output layer.

## 3. Results and Discussion

### 3.1. Identification of Isolated Compounds from D. crassirhizoma

**Figure 1:**
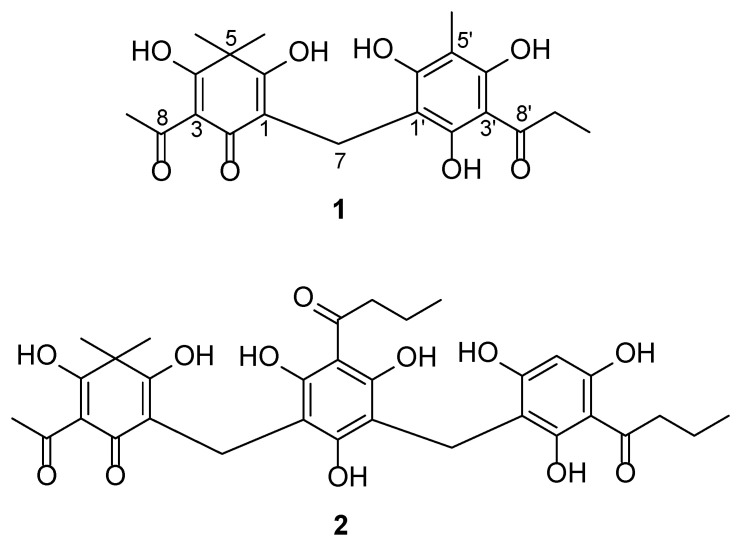
Chemical structure of flavaspidic acid AP (**1**) and nortrisflavaspidic acid ABB (**2**).

The ethanolic extract of *D. crassirhizoma* was partitioned into fractions and isolated by combined chromatographic methods, such as silica gel, Sephadex LH-20, and RP-18 CC, to obtain compounds **1** and **2** (Figure 1 and Figure S2–S7). The isolated compounds (**1** and **2**) were confirmed to be flavaspidic acid AP and nortrisflavaspidic acid ABB, respectively, by analyzing NMR spectroscopic data and comparison with the literature [10,11].

Physical and spectroscopic data of isolated compounds:

Flavaspidic acid AP (**1**): Yellow needles; ^1^H-NMR (CD_3_OD, 500 MHz): 3.46 (2H, s, H-7), 2.43 (3H, s, H-9), 1.28 (each 3H, s, H-11, H-12), 3.15 (2H, q, *J* = 7.3 Hz, H-9′), 1.15 (3H, t, *J* = 7.3 Hz, H-10′), 1.96 (3H, s, H-11′); ^13^C-NMR (CD_3_OD, 500 MHz): 108.7 (C-1), 184.1 (C-2), 105.5 (C-3), 202.7 (C-4), 53.6 (C-5), 197.2 (C-6), 18.6 (C-7), 194.2 (C-8), 26.6 (C-9), 25.4 (C-11, C-12), 106.3 (C-1′), 159.0 (C-2′), 106.0 (C-3′), 162.6 (C-4′), 104.4 (C-5′), 162.8 (C-6′), 208.7 (C-8′), 38.3 (C-9′), 9.6 (C-10′), 7.9 (C-11′).

Nortrisflavaspidic acid ABB (**2**): Off-white powder; ^1^H-NMR (CD_3_OD, 500 MHz): 3.47 (2H, s, H-7), 2.40 (3H, s, H-9), 1.23 (each 3H, s, H-11, H-12), 3.65 (2H, s, H-7′), 3.09 (2H, t, *J* = 7.3 Hz, H-9′), 1.66 (each 2H, m, H-10′, H-10″), 0.96 (each 3H, m, H-11′, H-11″), 5.83 (1H, br s, H-5″), 3.05 (2H, t, *J* = 7.3 Hz, H-9″); ^13^C-NMR (CD_3_OD, 500 MHz): 110.1 (C-1), 185.8 (C-2), 107.5 (C-3), 202.5 (C-4), 53.1 (C-5), 195.5 (C-6), 17.5 (C-7), 193.4 (C-8), 26.1 (C-9), 25.4 (C-11, C-12), 107.4 (C-1′), 160.1 (C-2′), 106.4 (C-3′), 162.1 (C-4′), 105.9 (C-5′), 164.0 (C-6′), 18.9 (C-7′), 208.6 (C-8′), 47.2 (C-9′), 19.4 (C-10′), 14.4 (C-11′), 107.0 (C-1″), 161.5 (C-2″), 106.1 (C-3″), 161.1 (C-4″), 97.2 (C-5″), 164.9 (C-6″), 207.9 (C-8″), 47.1 (C-9″), 19.4 (C-10″), 14.4 (C-11″).

### 3.2. Inhibitory Activity against α-Glucosidase

α-Glucosidase is an enzyme classified as EC 3.2.1.20 and is a member of the glycoside hydrolase family [20]. Primarily, it is found in epithelial cells of the small intestine and plays an important role in the breakdown of disaccharides and oligosaccharides into monosaccharides thereby facilitating carbohydrate digestion [22]. Inhibition of α-glucosidase is a potential therapeutic strategy for treating type 2 diabetes because it can slow the release of sugar from starch and oligosaccharides, thus leading to delayed sugar absorption and decreased postprandial blood sugar levels [23,24].

The inhibition of α-glucosidase by *D. crassirhizoma* was examined by investigating the inhibitory activities of different extract fractions (ethanol extract, CHCl_3_, EtOAc, and aqueous fractions) and two isolated compounds (**1** and **2**). The data presented in Table 1 show the inhibitory activity of four extract fractions against α-glucosidase. All four fractions exhibited inhibition at a concentration of 100 µg, with inhibition percentages ranging from 71.6 ± 1.39 to 79.9 ± 1.71. The IC_50_ value, which represents the concentration of the fraction required to inhibit α-glucosidase activity by 50%, was found to be 15.2 µg/mL for the EtOAc fraction, indicating that it is the most potent activity among all tested fractions. The EtOH extract, CHCl_3_, and aqueous fractions showed weaker inhibitory effects, with IC_50_ values of 34.8 ± 1.43, 25.8 ± 0.68, and 22.2 ± 1.92 µg/mL, respectively.

Additional examination of the isolated compounds demonstrated that at a concentration of 100 μM, only nortrisflavaspidic acid ABB (**2**) showed an inhibitory effect on α-glucosidase greater than 50%. Thus, this compound was tested at different concentrations to determine its IC_50_ value. Previous studies reported that trimeric phloroglucinol nortrisflavaspidic acid ABB (**2**) exhibited the significant inhibition of PTP1B and *β*-glucuronidase, with IC_50_ values of 1.19 ± 0.13 and 8.0 ± 1.8 μM, respectively [5,6]. In this study, nortrisflavaspidic acid ABB (**2**) demonstrated a strong inhibitory effect against α-glucosidase, with an IC_50_ value of 34.0 ± 0.14 μM, stronger than that of the positive control acarbose (IC_50_ = 329.2 ± 0.35 μM). Our results suggest that *D. crassirhizoma* could be a promising natural source of active compounds for the treatment of type 2 diabetes mellitus in general and inhibition of α-glucosidase in particular.

### 3.3. Development of the HPLC Analysis Method

**Figure 2:**
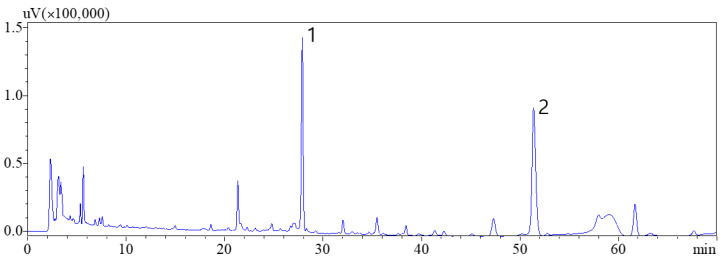
HPLC-UV chromatograph of *D. crassirhizoma* obtained at 280 nm. Flavaspidic acid AP (**1**) and nortrisflavaspidic acid ABB (**2**).

Various HPLC parameters were examined to develop a scientifically reliable and efficient method for quantifying the amounts of the two compounds in *D. crassirhizoma*. These parameters included the composition of the mobile phase (acetonitrile–water or methanol–water with formic acid and/or trifluoroacetic acid as buffers), the temperature of the column (25, 30, 35, and 40 °C), and flow rate of the mobile phase (0.6, 0.8, 1.0, and 1.2 mL/min). To obtain satisfactory resolution and separation, the UV spectra of the two components were characterized using the photodiode array detector. The optimal wavelength for detection was set to 280 nm. The optimized HPLC conditions resulted in good peak shape, separation, and resolution of the two components, indicating the suitability of this analytical method for the subsequent study using RSM (Figure 2).

### 3.4. RSM Optimization

Based on the results of our single-factor experiment, RSM with a BBD was conducted using Design-Expert 12 software to optimize three independent experimental parameters, including sonication time (A), sonication power (B), and the ratio of solvent (EtOH) to material (C). A total of 17 experiments were performed, and the BBD matrix for the three individual variables and response results (the sum peak area of compounds **1** and **2**) is presented in Table 2. The 17 designed experiments were conducted, the obtained data were statistically analyzed, and the results are presented in Table 3. In a statistical model, a higher F-value and a lower *p*-value indicate a more significant effect [25]. The F-value of 60.05 indicates that this model is significant. The model term is considered significant when its *p*-value is below 0.05. In this study, model terms A, B, AB, AC, BC, A^2^, B^2^, and C^2^ are significant. The f-value for the lack of fit is 5.67, indicating that the lack of fit is non-significant in comparison to the pure error. The *p*-value of 0.0634 indicates that there is only a 6.34% possibility that a lack of fit F-value could occur because of noise.

**Figure 3:**
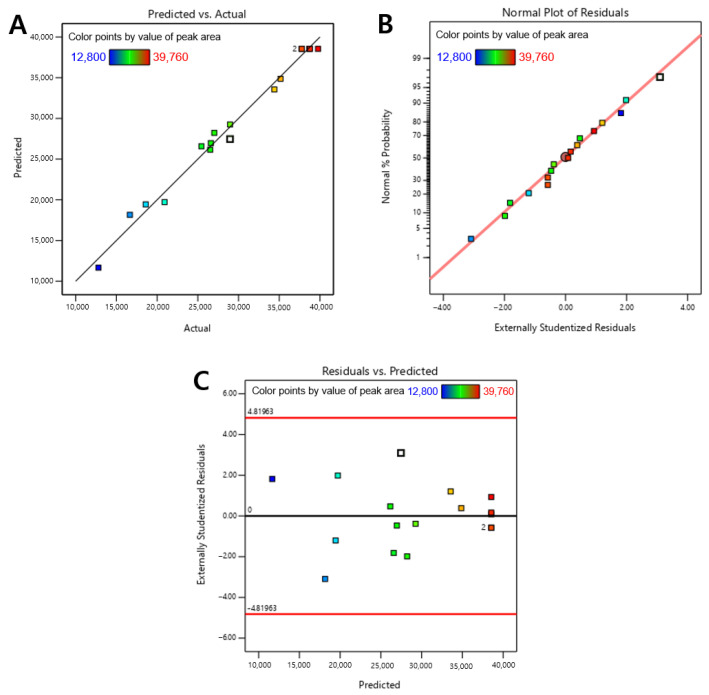
Plots of predicted vs. actual (**A**), normal percentage probability vs. externally studentized residuals (**B**), and externally studentized residuals vs. predicted response (**C**).

The adequacy of the developed model was assessed to evaluate the data analysis of the experiment. Figure 3A presents the predicted peak area of the active compounds, which agrees with the actual experimental results. The coefficient of determination is remarkably high, indicating a strong correlation between the predicted and actual values. As shown in Figure 3B, the normal percentage probability plot for externally studentized residuals of variables A, B, and C exhibits a uniform distribution with no variance, indicating that these individual variables behave well. The adjusted and simple coefficients of determination (Adjusted *R*^2^ and *R*^2^, respectively) are 0.9969 and 0.9872, respectively. These values indicate that the RSM model accounts for 99.69% of the variability of the corresponding variable, indicating that this model is a good fit for the experimental results. Therefore, the results suggest that the RSM model successfully describes the data and that the employed methodology is acceptable.

Equation (2) shows the relationship between the response and three individual variables:

Y = −34,1330.25 + 3376.25 × A + 1261.390625 × B − 191.96875 × C − 1.515625 × AB + 3.4375 × AC + 0.576719 × BC − 15.430625 × A^2^ − 1.691836 × B^2^ − 1.914219 × C^2^ (2)

where Y is the sum of the peak areas of two active compounds (**1** and **2**).

### 3.5. Combined Effect of Solvent Concentration, Power, and Extraction Time

**Figure 4:**
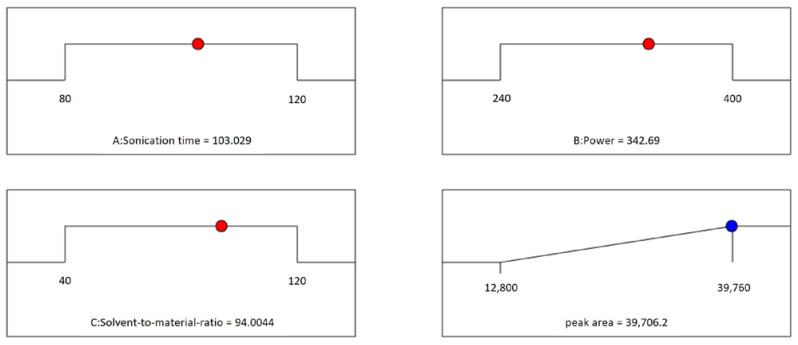
Desirability ramps for the three variables to obtain the maximum total amount of two compounds (desirability = 0.998, solution 1 out of 1).

The data points on each ramp shown in Figure 4 show the anticipated response of the best choice for extracting the maximum amount of the two compounds. The optimal settings are attained at the extraction time of 103.03 min, sonication power of 342.69 W, and solvent-to-material ratio of 94.00 mL/g.

**Figure 5:**
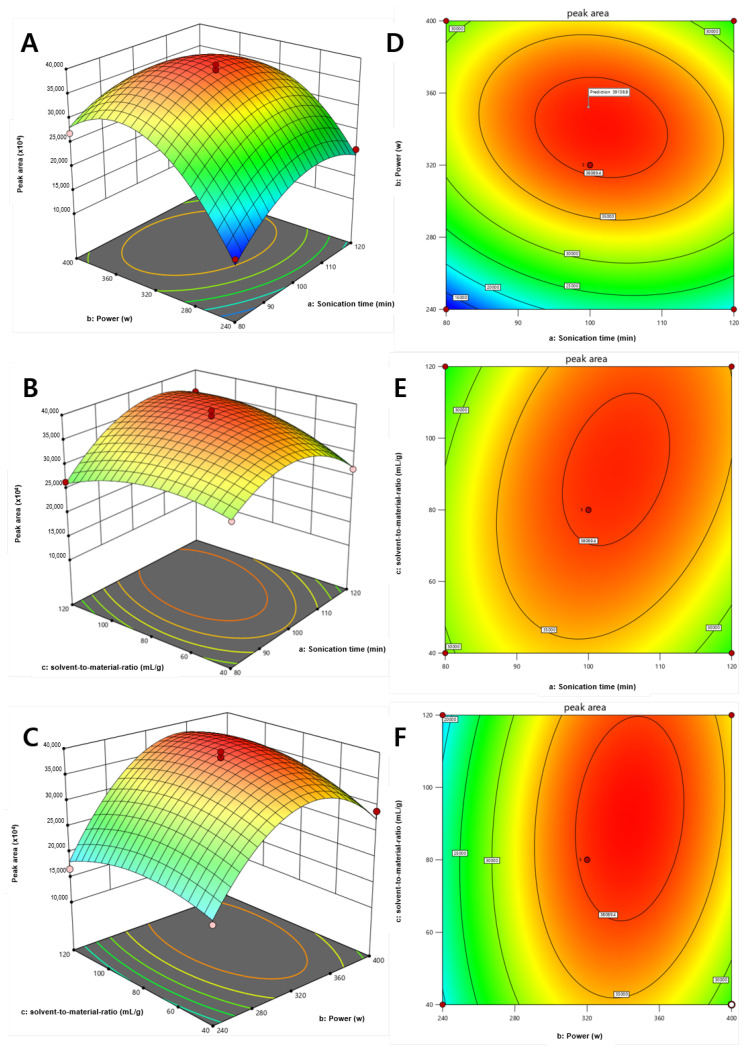
3D response surface graphs (**A**–**C**) and contour plots (**D**–**F**) of *D. crassirhizoma* under (**A**,**D**) solvent-to-material ration optimized to 94 mL/g, (**B**,**E**) sonication power optimized to 342.69 W, and (**C**,**F**) extraction time optimized to 103.03 min.

RSM was employed to examine the 3D response surface graphs, which were generated by examining the impact of three UAE properties on the sum of peak areas of two compounds, **1** and **2** (Figure 5). Figure 5A shows the effects of extraction time (A) and sonication power (B) on the total peak area of the two compounds at a constant solvent-to-material ratio. Sonication power was observed to have a weak effect on the yield of the two compounds. This observation is confirmed by the response surface plots shown in Figure 5C, which exhibit the impact of sonication power (B) and solvent-to-material ratio (C) on the yield of the two compounds. The plateau and curvature in Figure 5B,E show that the solvent-to-material ratio and the extraction time considerably affect extraction efficiency.

### 3.6. ANN

An ANN was used to confirm the RSM results. The ANN training algorithm used herein was trainlm, which is considered one of the fastest backpropagation algorithms in the toolbox for training the ANN [26]. The results of the comparative analysis of training, validation, and testing datasets, as well as a combined set of experimental and predicted data, are shown in Figure S8. The results demonstrate a high-level agreement between the predicted and experimental data, with all R values exceeding 0.985. The network was effectively trained, realizing a coefficient of determination of 0.99631. The regression coefficient (0.98637), which measures the relation between the output and objective, is close to 1, indicating excellent performance. Furthermore, the data predicted by the ANN agree with the experimental data, with minimal error. Therefore, this ANN model can be used to accurately predict the total amount of composites extracted from *D. crossirhizoma*.

### 3.7. Validation of the Optimal Conditions

To confirm the accuracy of the response equation and determine the optimal experimental parameters, the experiments were performed in triplicate, and the resulting data are presented in Table 4 and Table 5. Both the RSM and ANN models demonstrate high predictability of the UAE parameters, with the ANN model delivering more accurate predictions than the RSM model. The degree of similarity between the predicted and experimental values was analyzed, and the ANN model demonstrated a matching value of 97.51%, compared with that of 97.15% in the RSM model. The ANN technique can capture any type of nonlinearity, overcoming the limitations of RSM, and can be developed without a predefined experimental design. The RSM technique provides regression equations for prediction and identifies insignificant parameters or interaction factors, it reduces the complexity of the problem [27]. However, overall, the difference in matching values is slight; it indicates that both techniques can predict the yield of UAE with a high degree of accuracy (matching higher than 97%).

## 4. Conclusions

Flavaspidic acid AP (**1**) and nortrisflavaspidic acid ABB (**2**), the most representative compounds from the ethanolic extract of *D. crassirhizoma*, have been successfully isolated and purified. The inhibitory activity of two secondary metabolites isolated from *D. crassirhizoma* against α-glucosidase was evaluated via in vitro assays, and nortrisflavaspidic acid ABB demonstrated higher inhibitory activity, indicating that this compound may potentially be used as an α-glucosidase inhibitor. When combined with the results of the previous studies, the obtained data suggest that *D. crassirhizoma* is a promising source for the production of functional foods for treating type 2 diabetes. A sensitive and rapid HPLC-UV method has been successfully established to quantify the amounts of these compounds in *D. crassirhizoma*. The parameters of the ultrasonic extraction of these compounds were optimized using RSM and ANN. The optimal conditions determined using the RSM are the extraction time of 103.03 min, sonication power of 342.69 W, and solvent-to-material ratio of 94.00 mL/g. The results predicted by the ANN demonstrated a high level of agreement with the experimental data, with all R values higher than 0.985. The RSM and ANN models were also validated. The agreement between the predicted and experimental values was higher than 97%, indicating that both models may be employed for optimizing the industrial extraction of active compounds from *D. crassirhizoma*.

## Figures and Tables

**Table 1:** α-Glucosidase inhibitory activities of EtOH extract, fractions, and compounds.

Extract or Fraction	100 µg (%)	IC_50_ (µg/mL)	Compound	100 µM (%)	IC_50_ (µM)
Ethanol extract	73.8 ± 0.19	34.8 ± 1.43	1	<50	–
CHCl_3_ fraction	71.6 ± 1.39	25.8 ± 0.68	2	59.5 ± 1.00	34.0 ± 0.14
EtOAc fraction	77.7 ± 0.44	15.2 ± 0.57			
Aqueous fraction	79.9 ± 1.71	22.2 ± 1.92	Acarbose ^a^	21.7 ± 1.43	329.2 ± 0.35

^a^ Positive control; (–) Not tested.

**Table 2:** BBD matrix for three independent variables in coded form and the peak area in *D. crassirhizoma* (*n* = 3).

Run	A: Sonication Time (min)	B: Sonication Power (W)	C: Solvent-to-Material Ratio (mL/g)	Response Peak Area Y (AU/min, ×10^4^)
1	80 (−1)	240 (−1)	80 (0)	12,800
2	100 (0)	400 (+1)	40 (−1)	28,960
3	120 (+1)	240 (−1)	80 (0)	20,920
4	80 (−1)	320 (0)	40 (−1)	28,960
5	100 (0)	320 (0)	80 (0)	38,780
6	120 (+1)	400 (+1)	80 (0)	25,440
7	100 (0)	320 (0)	80 (0)	38,665
8	100 (0)	320 (0)	80 (0)	37,760
9	120 (+1)	320 (0)	120 (+1)	35,160
10	80 (−1)	320 (0)	120 (+1)	26,520
11	100 (0)	240 (−1)	120 (+1)	16,658
12	120 (+1)	320 (0)	40 (−1)	26,600
13	80 (−1)	400 (+1)	80 (0)	27,020
14	100 (0)	320 (0)	80 (0)	37,760
15	100 (0)	320 (0)	80 (0)	39,760
16	100 (0)	400 (+1)	120 (+1)	34,400
17	100 (0)	240 (−1)	40 (−1)	18,600

**Table 3:** Analysis of the variance of the BBD predicted model.

Source	Sum of Squares	df ^1^	Mean Square	F-Value ^2^	*p*-Value	Remarks
Model	1.127 × 10^9^	9	1.253 × 10^8^	60.05	<0.0001	significant
A–Sonication time	2.054 × 10^7^	1	2.054 × 10^7^	9.85	0.0164	
B–Power	2.743 × 10^8^	1	2.743 × 10^8^	131.48	<0.0001	
C–Solvent-to-material-ratio	1.156 × 10^7^	1	1.156 × 10^7^	5.54	0.0508	
AB	2.352 × 10^7^	1	2.352 × 10^7^	11.28	0.0121	
AC	3.025 × 10^7^	1	3.025 × 10^7^	14.50	0.0066	
BC	1.362 × 10^7^	1	1.362 × 10^7^	6.53	0.0378	
A²	1.604 × 10^8^	1	1.604 × 10^8^	76.90	<0.0001	
B²	4.936 × 10^8^	1	4.936 × 10^8^	236.65	<0.0001	
C²	3.950 × 10^7^	1	3.950 × 10^7^	18.93	0.0033	
Residual	1.460 × 10^7^	7	2.086 × 10^6^			
Lack of Fit	1.182 × 10^7^	3	3.941 × 10^6^	5.67	0.0634	not significant
Pure Error	2.778 × 10^6^	4	6.946 × 10^5^			
Cor Total ^3^	1.142 × 10^9^	16				
*R* ^2^	0.9872		Adjusted *R*^2^	0.9969		
C.V. %	4.9600		Predicted *R*^2^	0.9853		

^1^ df: degree of freedom; ^2^ F-value was calculated by dividing the mean square of the residual by the mean square of the source; ^3^ Cor total: the correlation total was calculated as the sum of squares and the degree of freedom.

**Table 4:** Three variables and the peak area in *D. crassirhizoma* comparison between actual value, predicted RSM, and predicted ANN (*n* = 3).

Run	Sonication Time (min)	Sonication Power (W)	Solvent-to-Material Ratio (mL/g)	Actual Value	Predicted (RSM)	Predicted (ANN)
1	80	240	80	12,800	11,662	11,164
2	100	400	40	28,960	27,462	29,371
3	120	240	80	20,920	19,717	19,638
4	80	320	40	28,960	29,255	29,008
5	100	320	80	38,780	38,545	37,478
6	120	400	80	25,440	26,578	24,788
7	100	320	80	38,665	38,545	37,478
8	100	320	80	37,760	38,545	37,478
9	120	320	120	35,160	34,865	33,546
10	80	320	120	26,520	26,160	26,283
11	100	240	120	16,658	18,156	14,389
12	120	320	40	26,600	26,960	30,494
13	80	400	80	27,020	28,223	27,084
14	100	320	80	37,760	38,545	37,478
15	100	320	80	39,760	38,545	37,478
16	100	400	120	34,400	33,558	34,557
17	100	240	40	18,600	19,443	17,434

**Table 5:** Predicted and experimental values of the response obtained under optimal conditions (*n* = 3).

	A (min)	B (W)	C (mL/g)	Y (AU/min, ×10^4^)
Predicted (RSM)	103.03	342.69	94.00	39,706.2
Predicted (ANN)	103.0	340.0	94.0	39,562.1
Experimental	103.0	340.0	94.0	38,575.2
Matching (RSM, %)				97.15%
Matching (ANN, %)				97.51%

## Data Availability

Data that support the findings of this study are available in the Article and the Supplementary Materials.

## Supplementary Materials

The following supporting information can be downloaded at: https://www.mdpi.com/article/10.3390/metabo13040557/s1, Figure S1: Selection of variables for RSM optimization; Figure S2: The HPLC-UV chromatographs of DC with flavaspidic acid AP (**1**); Figure S3: The HPLC-UV chromatographs of DC with nortrisflavaspidic acid ABB (**2**); Figure S4: ESI-MS spectrum (positive mode and negative mode) of flavaspidic acid AP (**1**); Figure S5: ^1^H-NMR spectrum (CD_3_OD, 500 MHz) of nortrisflavaspidic acid ABB (**2**); Figure S6: ^13^C-NMR spectrum (CD_3_OD, 125 MHz) of nortrisflavaspidic acid ABB (**2**); Figure S7: HR-ESI-MS spectrum (negative mode) of nortrisflavaspidic acid ABB (**2**); Figure S8: The prediction graphs for them training, validation, and test of the established network.
