# Supplementary material for: Optimization of Ultrasonic-Assisted Extraction of α-Glucosidase Inhibitors from Dryopteris crassirhizoma Using Artificial Neural Network and Response Surface Methodology
Source: Metabolites. 2023 Apr 13;13(4):557. doi: 10.3390/metabo13040557 (PMC10145310; doi:10.3390/metabo13040557)
Supplement: Supplementary file 1 [file metabolites-13-00557-s001.zip › metabolites-2323328-supplementary.pdf]

Supplemental material

Optimization of ultrasonic-assisted extraction of *α*-glucosidase inhibitors from *Dryopteris crassirhizoma* using artificial neural network and response surface methodology

Nguyen Viet Phong [1,2,†], Dan Gao [3,†], Jeong Ah Kim [1,2,*], Seo Young Yang [4,*]

[1] *Vessel-Organ Interaction Research Center, VOICE (MRC), College of Pharmacy, Kyungpook National University, Daegu 41566, Korea; ngvietphong@gmail.com (N.V.P.), jkim6923@knu.ac.kr (J.A.K.)*

[2] *BK21 FOUR Community-Based Intelligent Novel Drug Discovery Education Unit, College of Pharmacy and Research Institute of Pharmaceutical Sciences, Kyungpook National University, Daegu 41566, Korea*

[3] *Institute of Chinese Materia Medica, China Academy of Chinese Medical Sciences, Beijing 100700, China; dgao@icmm.ac.cn (D.G.)*

[4] *Department of Pharmaceutical Engineering, Sangji University, 83 Sangjidae-gil, Wonju 26339, Korea; syyang@sangji.ac.kr (S.Y.Y.)*

* Correspondence: jkim6923@knu.ac.kr (J.A.K.); syyang@sangji.ac.kr (S.Y.Y.); Tel.: +82-53-950-8574 (J.A.K); +82-33-738-7921 (S.Y.Y.)

† These authors contributed equally to this work.

Figure S1. Selection of variables for RSM optimization

Figure S2. The HPLC-UV chromatographs of DC with flavaspidic acid AP (**1**)

Figure S3. The HPLC-UV chromatographs of DC with nortrisflavaspidic acid ABB (**2**)

Figure S4. ESI-MS spectrum (positive mode and negative mode) of flavaspidic acid AP (**1**)

Figure S5. ^{1}H-NMR spectrum (CD_3OD, 500 MHz) of nortrisflavaspidic acid ABB (**2**)

Figure S6. ^{13}C-NMR spectrum (CD_3OD, 125 MHz) of nortrisflavaspidic acid ABB (**2**)

Figure S7. HR-ESI-MS spectrum (negative mode) of nortrisflavaspidic acid ABB (**2**)

Figure S8. The prediction graphs for them training, validation, and test of the established network

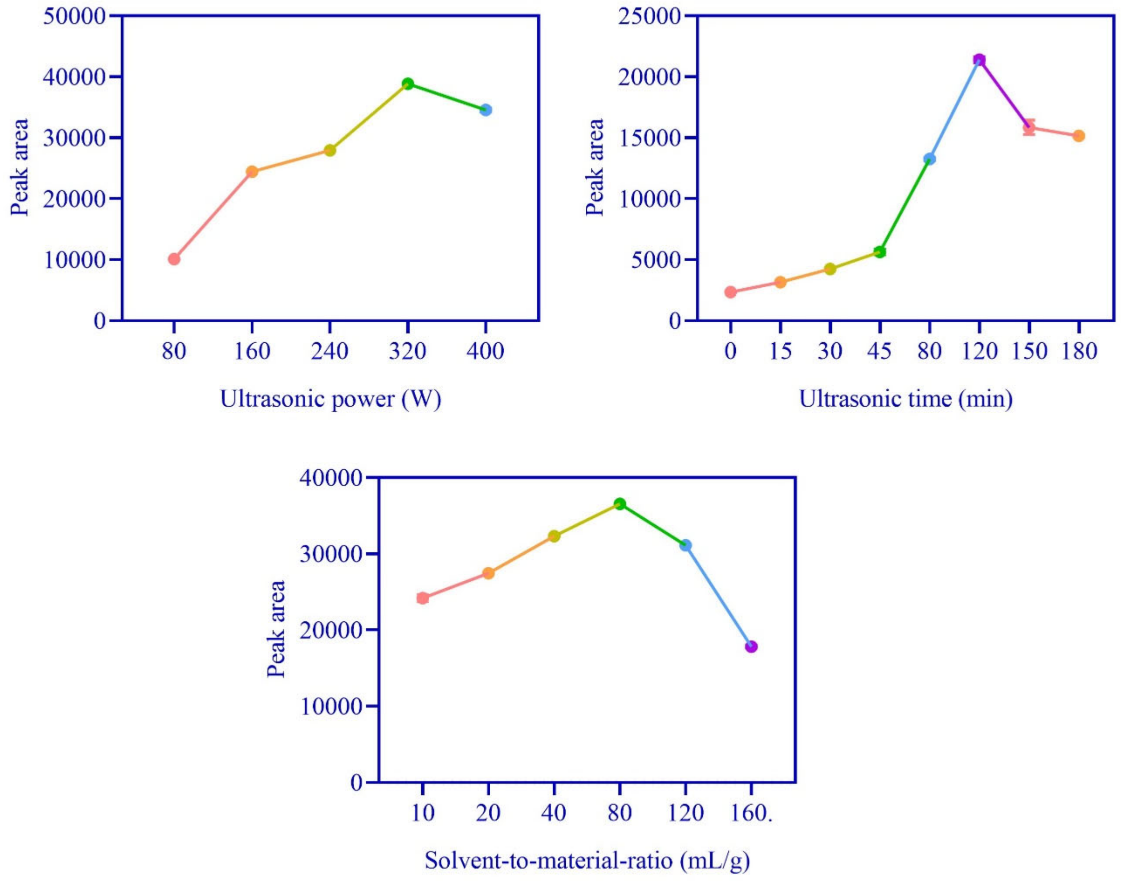

Figure S1. Selection of variables for RSM optimization

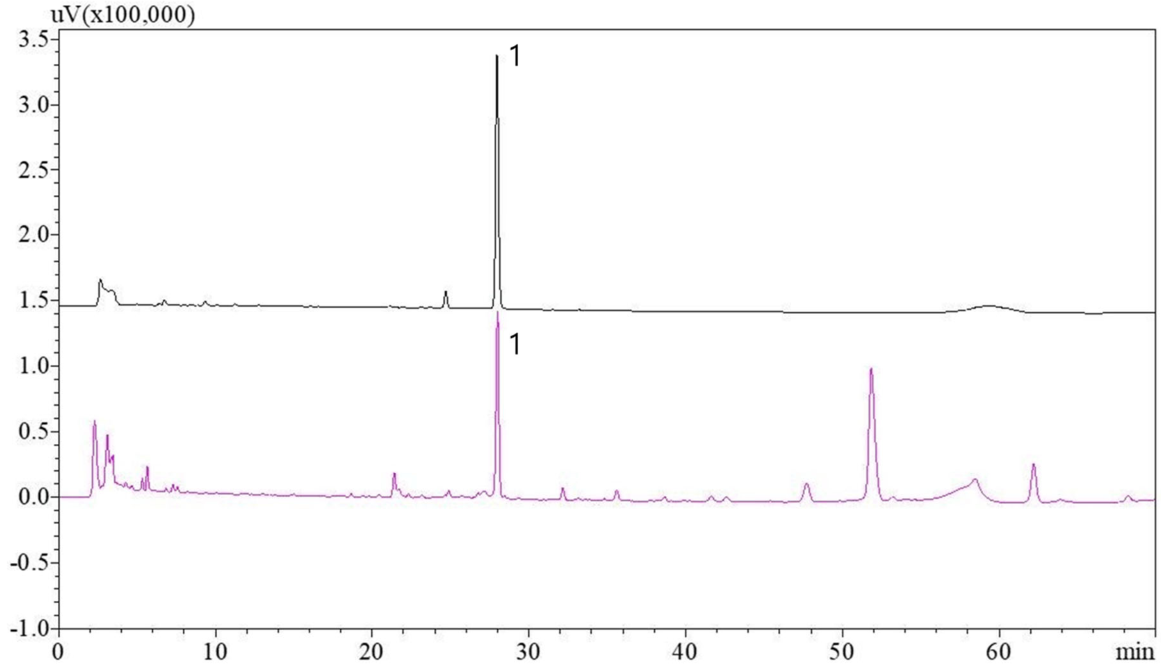

Figure S2. The HPLC-UV chromatographs of DC with flavaspidic acid AP (**1**)

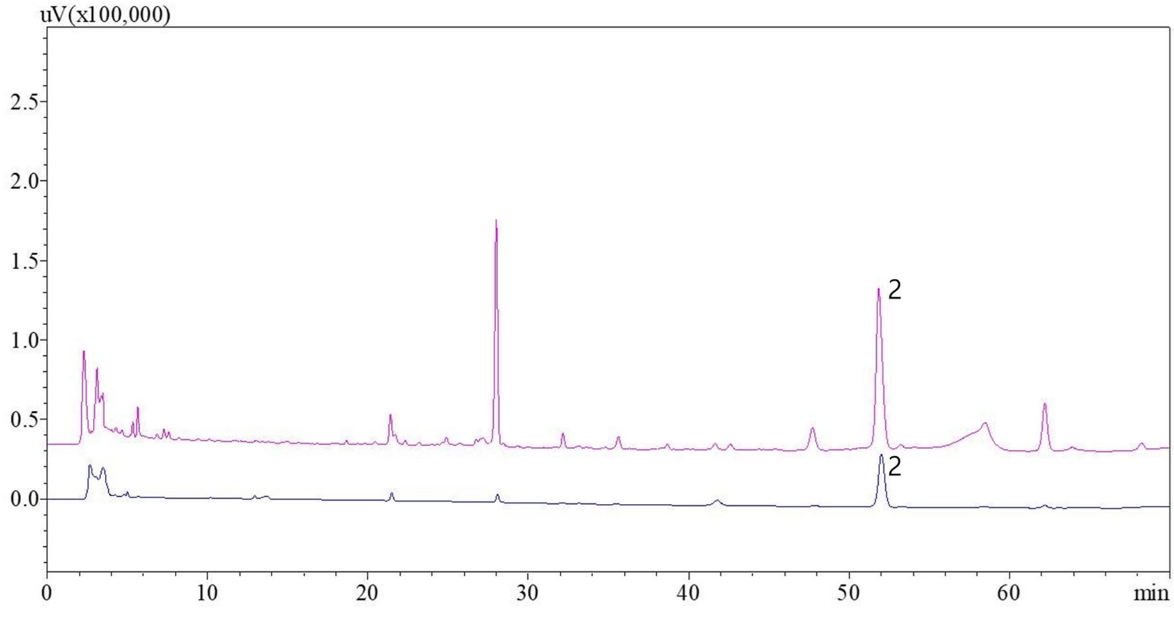

Figure S3. The HPLC-UV chromatographs of DC with nortrisflavaspidic acid ABB (**2**)

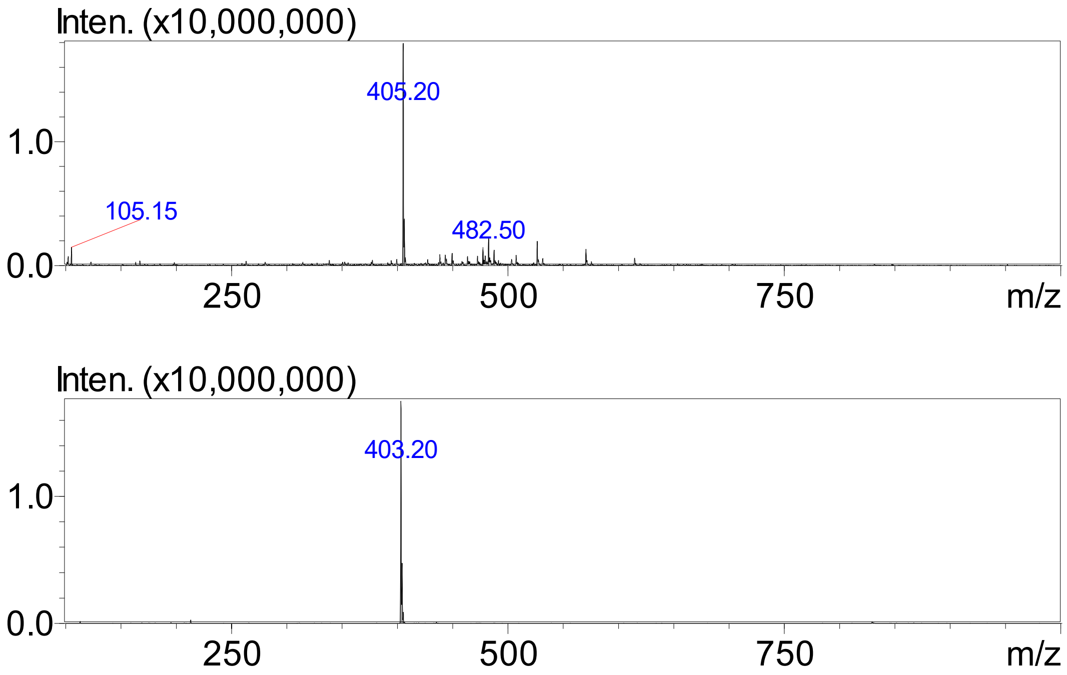

Figure S4. ESI-MS spectrum (positive mode and negative mode) of flavaspidic acid AP (**1**)

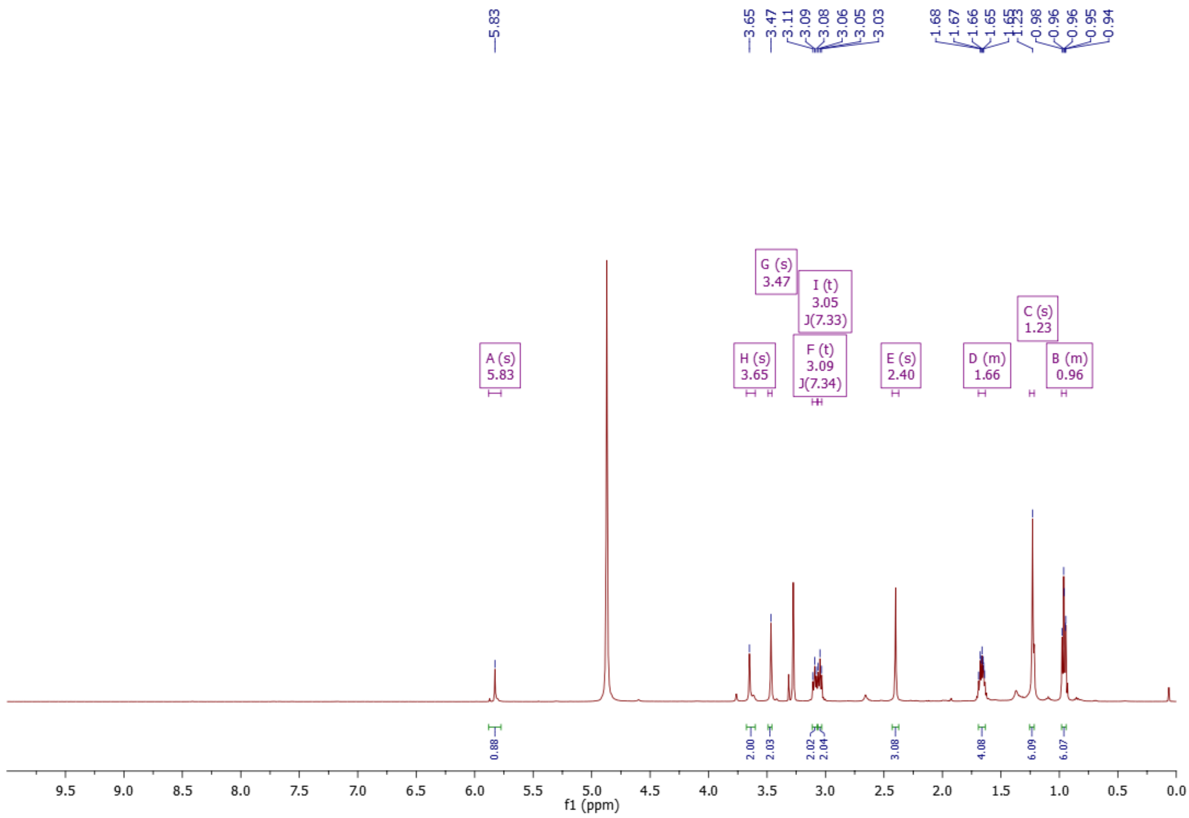

Figure S5. ^{1}H-NMR spectrum (CD_3OD, 500 MHz) of nortrisflavaspidic acid ABB (**2**)

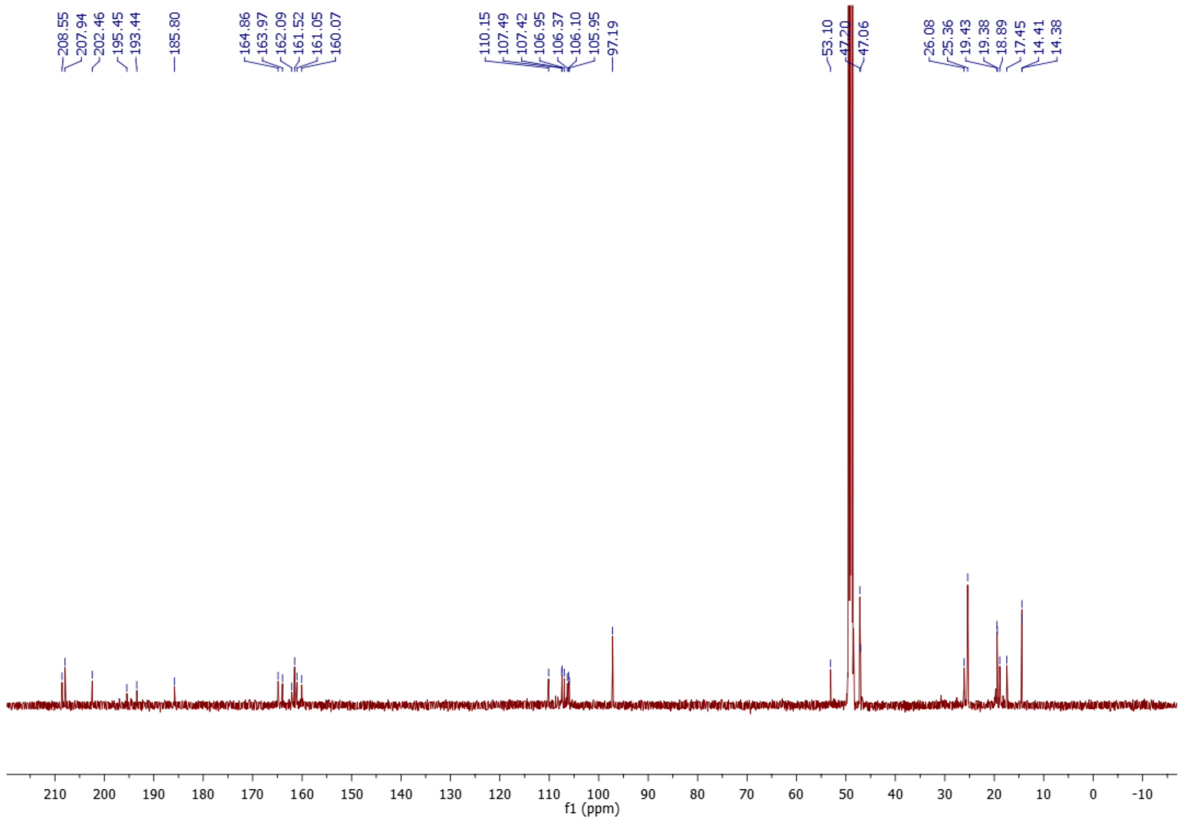

Figure S6. ^{13}C-NMR spectrum (CD_3OD, 125 MHz) of nortrisflavaspidic acid ABB (**2**)

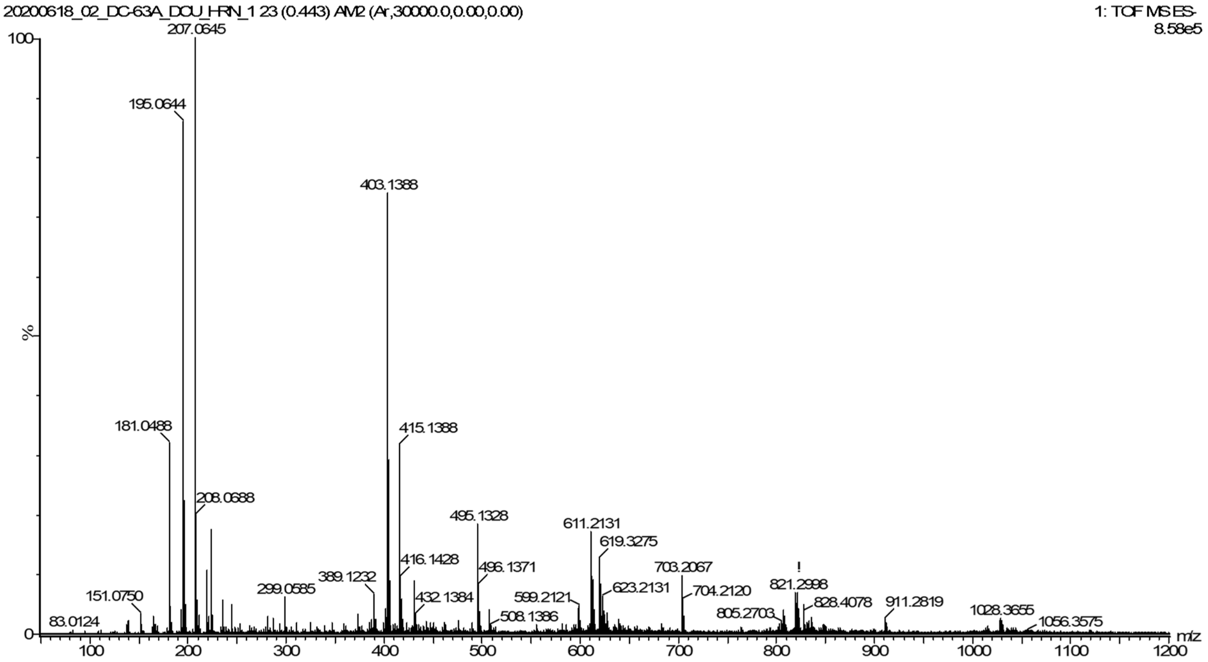

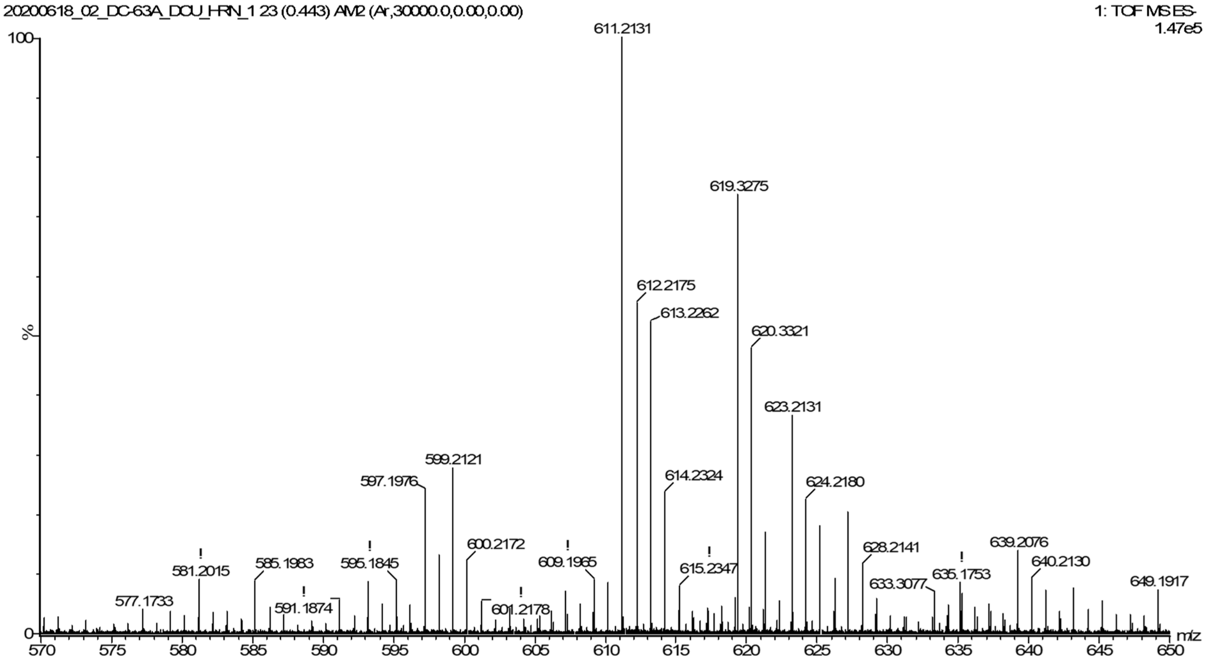

Elemental Composition Report

Single Mass Analysis
Tolerance = 5.0 PPM / DBE: min = -1.5, max = 100.0
Element prediction: Off
Number of isotope peaks used for i-FIT = 3

Monoisotopic Mass, Even Electron Ions
27 formula(e) evaluated with 1 results within limits (all results (up to 1000) for each mass)
Elements Used:
C: 0-40 H: 0-45 O: 0-12

Minimum:				-1.5				
Maximum:		100.0	5.0	100.0				
Mass	Calc. Mass	mDa	PPM	DBE	i-FIT	Norm	Conf(%)	Formula
611.2131	611.2129	0.2	0.3	15.5	654.2	n/a	n/a	C32 H35 O12

Figure S7. HR-ESI-MS spectrum (negative mode) of nortrisflavaspidic acid ABB (**2**)

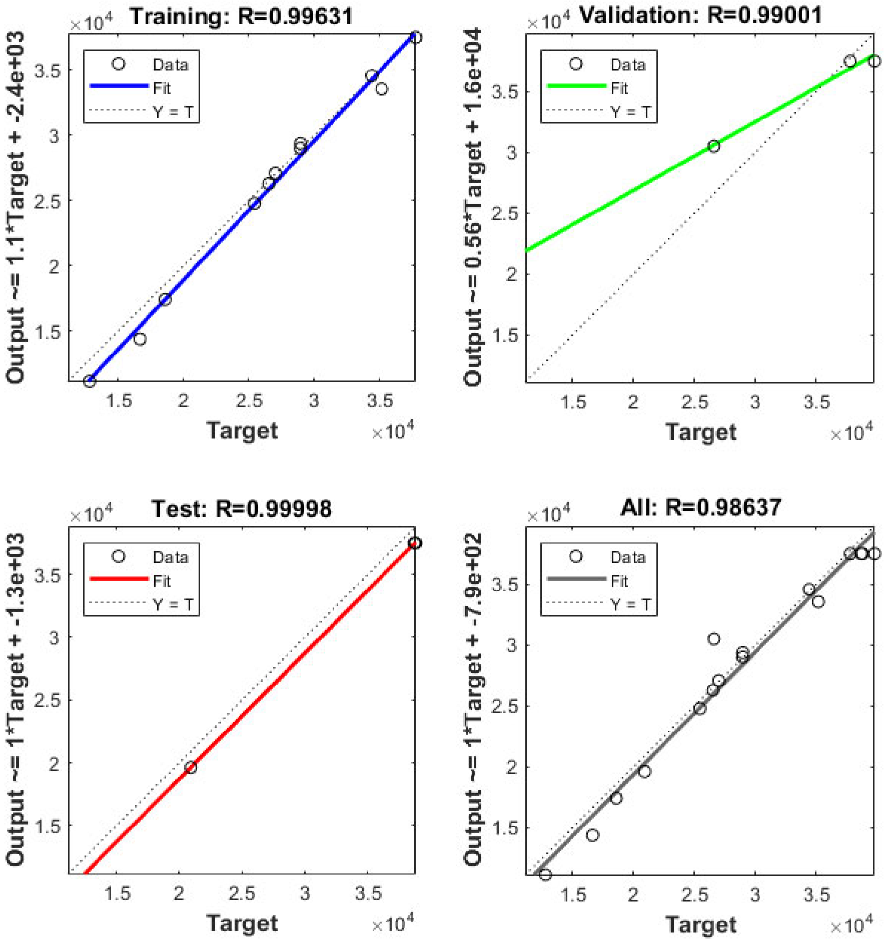

Figure S8. The prediction graphs for them training, validation, and test of the established network
